# Clinical efficacy of diode laser for pulpotomy in primary teeth: a meta-analysis of randomised controlled trials

**DOI:** 10.2340/aos.v84.43804

**Published:** 2025-06-10

**Authors:** Yue Gao, Mina Hu, Jian Xu

**Affiliations:** a Department of Periodontology, Shaoxing Stomatological Hospital, Shaoxing City, China; b Department of Endodontics, Shaoxing Stomatological Hospital, Shaoxing City, China

**Keywords:** diode laser, formocresol, pulpotomy, meta-analysis, randomized controlled trials

## Abstract

**Objective:**

To systematically evaluate the efficacy of diode laser for pulpotomy in primary teeth using meta-analysis.

**Methods:**

Electronic databases (PubMed, Web of Science, Cochrane Library, and Embase) were systematically searched to include randomised controlled trials (RCTs) evaluating the efficacy of diode laser pulpotomy in primary teeth, with the control group receiving conventional treatment without diode laser. This meta-analysis was based on a systematic literature search. Meta-analyses were conducted based on different follow-up times and control groups to assess the clinical and radiographic success rates of diode laser pulpotomy.

**Results:**

A total of 17 RCTs met the inclusion criteria, involving 1,018 primary teeth. Meta-analysis results based on different follow-up times (≤ 3 months, 6 months, 9 months, ≥12 months) showed similar clinical success rates (total: relative risk [RR], 1.01; 95% confidence interval [CI], 0.99–1.03; *I*^2^ = 0%) and radiographic success rates (total: RR, 0.99; 95% CI, 0.97–1.02; *I*^2^ = 0%) between diode laser pulpotomy and conventional treatment. Meta-analysis based on different control groups (formocresol-zinc oxide eugenol [FC-ZOE], ferric sulfate-zinc oxide eugenol [FS-ZOE], mineral trioxide aggregate-zinc oxide eugenol [MTA-ZOE], simvastatin gel-resin modified glass ionomer cement [SG-REGIC]) showed similar clinical success rates between diode laser pulpotomy and FC-ZOE (RR: 1.00; 95% CI: 0.98–1.02), MTA-ZOE (RR: 1.01; 95% CI: 0.94–1.09), and SG-REGIC (RR: 0.99; 95% CI: 0.83–1.17), but it was more likely to achieve clinical success compared to FS-ZOE (RR: 1.04; 95% CI: 1.01–1.07). In addition, diode laser pulpotomy showed similar radiographic success rates with different control groups.

**Conclusion:**

Diode laser can be considered as an alternative treatment method to current conventional pulpotomy. However, further high-quality trials are needed to confirm the accuracy and reliability of these findings.

## Introduction

Pulpotomy is a surgical procedure used to treat deep caries exposure, traumatic exposure, or partial pulp infection in primary teeth. When direct pulp capping is not feasible, this surgery involves the removal of coronal pulp tissue under local anaesthesia and the application of medication to the pulp wound to preserve the healthy pulp tissue in the root [1, 2]. Commonly used medications include formocresol (FC), ferric sulfate (FS), and mineral trioxide aggregate (MTA). FC was once considered the ‘gold standard’ for pulpotomy in primary teeth [3], but its clinical application is controversial due to the cytotoxicity, teratogenicity, and carcinogenicity of formaldehyde and cresol in its composition [4]. FS solution forms an iron-protein complex upon contact with blood, which mechanically seals the cut vessels, achieving haemostasis and reducing pulp infection and root resorption [5]. However, it has high technical requirements and similar toxicity to FC, gradually being replaced by other drugs with better biocompatibility. MTA, because of its excellent marginal adaptation, ability to induce pulp cell proliferation, and formation of high-quality hard tissue barriers, has become the mainstream choice [6]. However, its high cost and strict storage requirements limit its widespread clinical application [7].

In recent years, one of the most innovative and popular advancements in the field of dentistry has been the application of laser technology, including CO_2_, diode, Nd: YAG, and Erbium lasers, which are commonly used for pulp ablation. Among them, diode laser has received increasing attention in pulpotomy because of its excellent haemostatic effect, strong antibacterial ability, and promotion of wound healing [8, 9]. Diode laser is mainly composed of aluminium, gallium, and arsenic, three excitation elements, and is an efficient laser device. Compared with traditional laser devices, diode laser instruments have the advantages of small size, high cost-performance ratio, and easy operation, and have been widely used in oral medicine. According to literature review, we have observed the efficacy of various techniques and drugs used in pulp ablation. Previous studies have shown that the success rate of diode laser pulpotomy is significantly higher than that of FC pulpotomy [10, 11], but some scholars have also observed contradictory results [12]. Due to inconsistent clinical, radiological, and histo-pathological results, the clinical efficacy of diode laser pulpotomy still needs further confirmation. Therefore, this study aims to comprehensively evaluate the therapeutic effect and safety of diode laser in pulpotomy of primary teeth through meta-analysis, providing evidence-based medical basis for clinical decision-making.

## Materials and methods

### Search strategy

This meta-analysis was based on a systematic literature search. Following the Preferred Reporting Items for Systematic Reviews and Meta-Analyses (PRISMA) 2020 statement [13], a systematic search without any filters and limits was conducted in four electronic databases: PubMed, Web of Science, Cochrane Library, and Embase. The search period was from the inception of the database to December 30, 2024. The main search strategy was as follows: (‘ablation, laser’ [MeSH Terms] OR ‘laser’ OR ‘diode laser’) AND (‘pulpotomy’ [MeSH Terms] OR ‘pulpotomy’) AND (‘primary teeth’ [MeSH Terms] OR ‘deciduous teeth’ [MeSH Terms] OR ‘primary molars’). In addition, target literature was obtained by reviewing the references of the included studies. This study has been registered in https://inplasy.com/ with the registration number INPLASY202540034.

### Inclusion and exclusion criteria

Inclusion criteria: (1) Studies published in peer-reviewed journals in Chinese and English; (2) Pulpotomy in primary teeth using diode laser; (3) The control group receives conventional treatment without diode laser; (4) Reports on the number of effective and ineffective teeth evaluated by clinical or radiographic examination results; (5) The study design is a randomised controlled trial (RCT).

Exclusion criteria: (1) Non-human studies; (2) Observational studies, conference articles, case reports, systematic reviews, and other types of studies; (3) Insufficient outcome information and inability to perform data analysis; (4) Duplicate reporting of literature studies; (5) Studies for which the full text cannot be obtained.

### Literature screening and data extraction

Two researchers independently screened the literature according to the inclusion and exclusion criteria. Initially, the literature was screened by reading the titles and abstracts, and then the full text of potentially eligible studies was read. When there were disagreements between the two researchers, a third researcher was consulted to reach a consensus. After the literature screening was completed, two researchers independently extracted data according to the standard data extraction form, extracting information including literature information, demographic characteristics of the study subjects, tooth characteristics, diode laser information, study time, and outcome events. In case the information was missing, the researchers were contacted for the missing information. If they provided the data, then the study would be included, or it would be excluded. If the data were ratio, we would calculate the event numbers according to ratio and sample size.

### Quality evaluation

The Cochrane collaboration risk assessment tool (ROB1.0) [14] was used to evaluate the quality of the literature. This method evaluates aspects such as random allocation methods, allocation concealment, blinding, completeness of outcome data, selective reporting of study results, and other sources of bias.

### Statistical analysis methods

Revman5.3 software was used for statistical analysis. The effect size of the count data was represented by relative risk (RR), and the 95% confidence interval (CI) was used to estimate the range of the effect size. Heterogeneity was judged by *I*^2 statistics and *Q* test, with *I*^2<50% or *p* > 0.1 considered to have homogeneity among the included studies, and a fixed-effect model was used for analysis; if *I*^2>50% or *p* ≤ 0.1, it was considered that the homogeneity among the included studies was poor, and a random-effect model was used for analysis. Subgroup analysis was conducted based on different follow-up times and different control groups. Funnel plots were used to determine whether there was publication bias among the included studies. If heterogeneity was large, sensitivity analysis was conducted to explore the source of heterogeneity. Unless otherwise specified, the test level was set to 0.05.

## Results

### Characteristics of included studies

**Figure 1:**
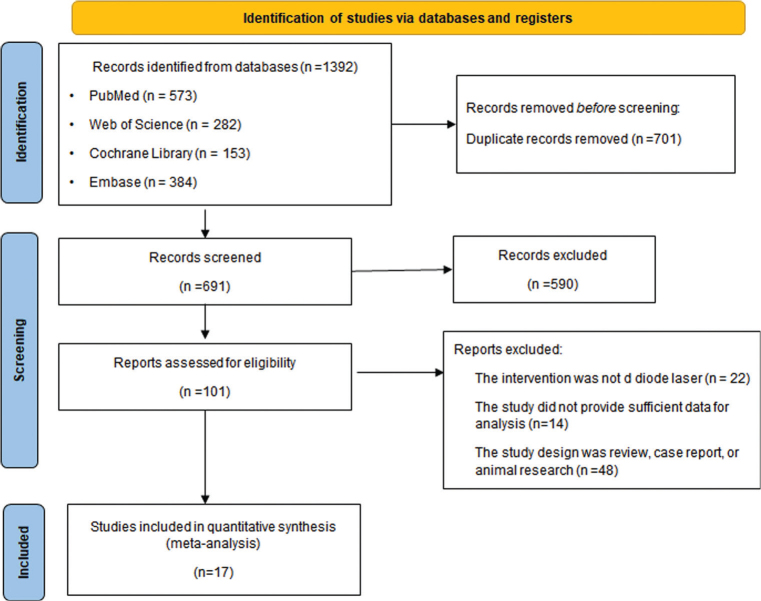
Flowchart of study screening.

After searching public electronic databases, a total of 1,392 studies were included in the literature review process, and the literature screening process is shown in Figure 1. After excluding 701 duplicate studies and 590 irrelevant studies, 101 studies were reviewed in full, and ultimately 17 qualified studies were included [10–12, 15–28].

Table 1 summarises the basic characteristics of the included studies. The publication time of the included studies ranged from 2005 to 2022, with studies from India (*n* = 8), Iran (*n* = 4), Brazil (*n* = 1), Canada (*n* = 1), Taiwan, China (*n* = 1), Egypt (*n* = 1), and Turkey (*n* = 1). All study subjects were children, aged between 2 and 10 years, involving 1,018 primary teeth. In terms of intervention, all studies used diode lasers, and the control groups included FC, FS, MTA, and simvastatin gel (SG). Zinc oxide eugenol (ZOE) was the commonly used pulp capping material. In different studies, the final restorative material for pulpotomy was mainly stainless steel crowns (SSC), with one study using resin-modified glass ionomer cement (RMGIC).

**Table 1:** Basic information of included studies.

Study	Location	Study design	Population	Mean age	Sample	Intervention	Pulp capping agent	Final restoration	Comparison
Saltzman B, 2005	Canada	RCT	Children aged 3–8	5.1 ± 1.2	26	**Diode laser** 3w, 980 nm, 0·5 mm diameter optical fibre, continuous pulse, contact	MTA	SSC	FC-ZOE
Golpayegani MV, 2009	Iran	RCT	Children aged 4–7 years	4–7	36	**Diode laser** 632 nm, 4 J/cm^2^, 0.5 mm-diameter optical fibre non-contact	ZOE	SSC	FC-ZOE
Durmus B, 2014	Turkey	RCT	Children aged 5–9 year	5–9	120	**Diode laser** 1.5w, 810 nm, 50 mJ, 30 Hz, noncontact	ZOE	SSC	FC-ZOEFS-ZOE
Yadav P, 2014	India	RCT	Children aged 4–7 years	4–7	45	**Diode laser** 3w, 810 nm, 400 mm-diameter optical fibre, continuous mode, non-contact	ZOE	SSC	FS-ZOE
Fernandes AP, 2015	Brazil	RCT	Children aged 5–9 year	6	30	**Diode laser** 660 nm, 10 mW, 300 mm-diameter optical fibre, 2.5 J/cm^2^, 50–60 Hz, 0.04 cm^2^, contact	ZOE	RMGIC	FC-ZOE
Niranjani K, 2015	India	RCT	Children aged 5–9 year	5–9	40	**Diode laser** 1.5w, 810 nm, 50 mJ, pulsed mode, contact	ZOE	SSC	MTA-ZOE
Gupta G, 2015	India	RCT	Children aged 4–10 year	4–10	20	**Diode laser** 980 nm, 4 J/cm^2^, 3 W, 0.5 mm diameter optical fibre, continuous pulsed mode, contact	ZOE	SSC	FS-ZOE
Uloopi KS, 2016	India	RCT	Children aged 4–7 years	4–7	40	**Diode laser** 810 nm, 2 J/cm^2^, 10 mW, continuous, non-contact	ZOE	SSC	MTA-ZOE
Joshi P, 2017	India	RCT	Children aged 4–9 year	4–9	38	**Diode laser** 980 nm, 1.5 W, 200 mm optical fibre tip, contact	ZOE	SSC	FC-ZOE
Ansari G, 2018	Iran	RCT	Children aged 3–9 years	3–9	120	**Diode laser** 980 nm, 1.5 W, 200 mm optical fibre tip, contact	ZOE	SSC	FC-ZOEFS-ZOE
Ansari G, 2018	Iran	RCT	Children aged 3–9 years	6.4	40	**Diode laser** 810 nm, 800-μm fibre optic tip, 10w, 20H, non-contact	ZOE	SSC	FC-ZOE
Pei SL, 2020	China, Taiwan	RCT	Children aged 2–8 years	4.61	90	**Diode laser** 915 nm, 2 W, 100 Hz, 300 μm optical fibre, contact	ZOE	SSC	FC-ZOE
Alamoudi N, 2020	Egypt	RCT	Children aged 5–8 year	6.18	102	**Diode laser** 810 nm, 6.7J/cm^2^, 200-μm fibre optic tip, pulsed mode, non-contact	ZOE	SSC	FC-ZOE
Satyarth S, 2021	India	RCT	Children aged 6–8 year	7	40	**Diode laser** 1.5w, 810 nm, 200-μm fibre optic tip, contact	ZOE	SSC	MTA-ZOE
Aripirala M, 2021	India	RCT	Children aged 4–8 years	6.05	100	**Diode laser** 940 nm, 2 W, 4J/cm^2^, 300 mm optical fibre tip, contact	RMGIC	SSC	SG-RMGIC
Ebrahimi M, 2022	Iran	RCT	Children aged 4–7 years	5.5	63	**Diode laser** (1) 660 nm, 200 mW, continuous wave mode, contact; (2) 810 nm, 1 W, continuous wave mode, 300 mm optical fibre tip, contact	MTA	SSC	MTA-ZOE
Yavagal CM, 2021	India	RCT	Children aged 4–7 years	4–7	68	**Diode laser** 660 nm, 36 mW, non-contact	ZOE	SSC	FC-ZOE

RCT: randomised controlled trial; MTA: mineral trioxide aggregate; ZOE: zinc oxide eugenol; RMGIC: resin modified glass ionomer cement; SSC: stainless steel crown; FC: formocresol; FS: ferric sulfate; SG: simvastatin gel.

### Quality assessment of literature

**Figure 2:**
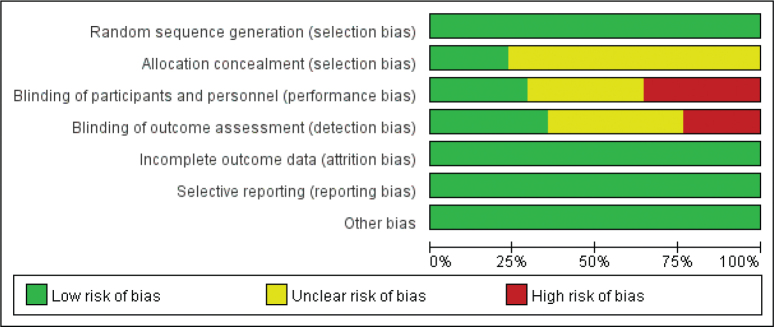
Risk of bias graph.

**Figure 3:**
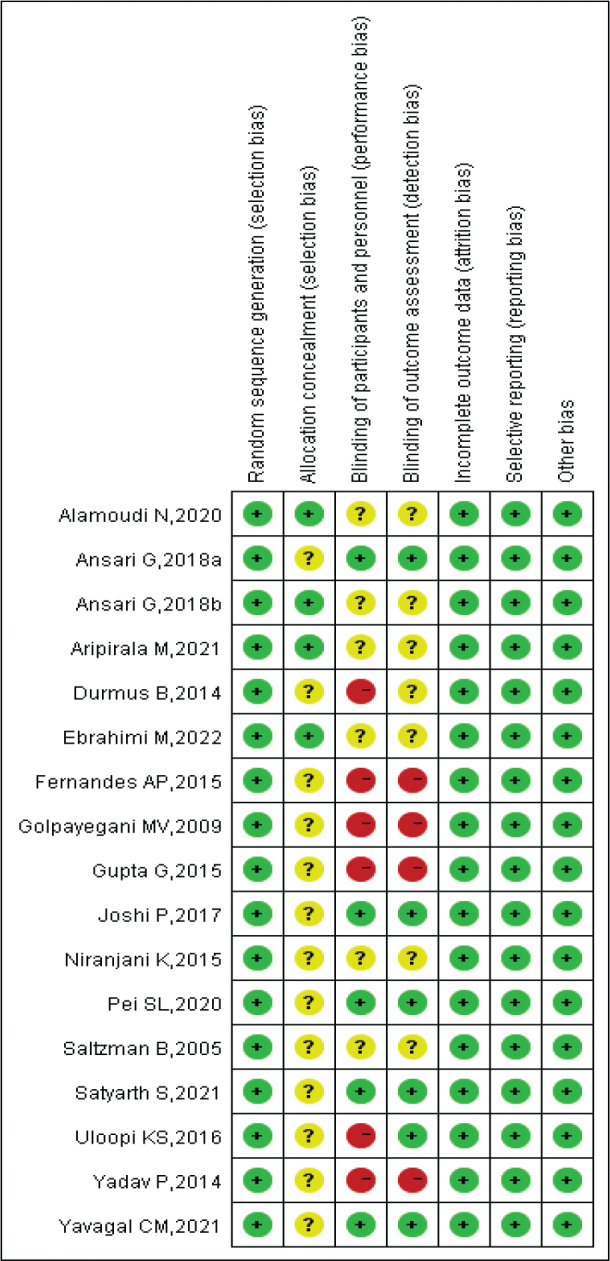
Risk of bias summary.

The quality of the RCT studies was assessed using the Cochrane collaboration risk of bias tool. The results showed that the included studies had certain biases in allocation concealment and blinding implementation, while they had lower biases in randomisation implementation, completeness of outcome data, selective reporting of study results, and other biases. Overall, the included studies had acceptable quality (Figures 2 and 3).

### Clinical success rate by different follow-up duration

**Figure 4:**
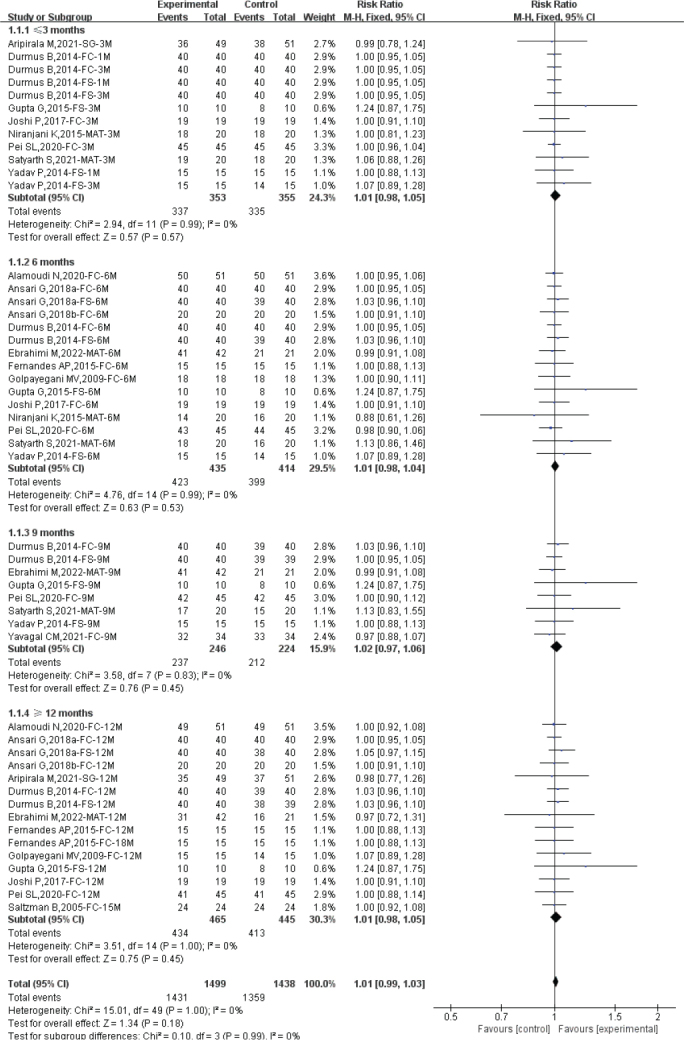
Forest plot comparing clinical success between diode laser and control by different follow-up duration.

We evaluated the clinical success rate of diode laser pulpotomy based on different follow-up times. The heterogeneity assessment showed good homogeneity among the included studies (Total: *I*^2^ = 0%; ≤3 months: *I*^2^ = 0%; 6 months: *I*^2^ = 0%; 9 months: *I*^2^ = 0%; ≥12 months: *I*^2^ = 0%), and a fixed-effect model was used for meta-analysis. The overall analysis suggested that diode laser pulpotomy had a similar clinical success rate compared to the control group (RR: 1.01; 95% CI: 0.99–1.03). For different follow-up times, diode laser pulpotomy showed similar clinical success rates compared to the control group at ≤3 months (*n* = 672; RR: 1.01; 95% CI: 0.98–1.05), 6 months (*n* = 822; RR: 1.01; 95% CI: 0.98–1.04), 9 months (*n* = 449; RR: 1.02; 95% CI: 0.97–1.06), and ≥12 months (*n* = 847; RR: 1.01; 95% CI: 0.98–1.05), indicating similar short-term, medium-term, and long-term clinical success rates for diode laser pulpotomy (see Figure 4).

### Radiographic success rate by different follow-up duration

**Figure 5:**
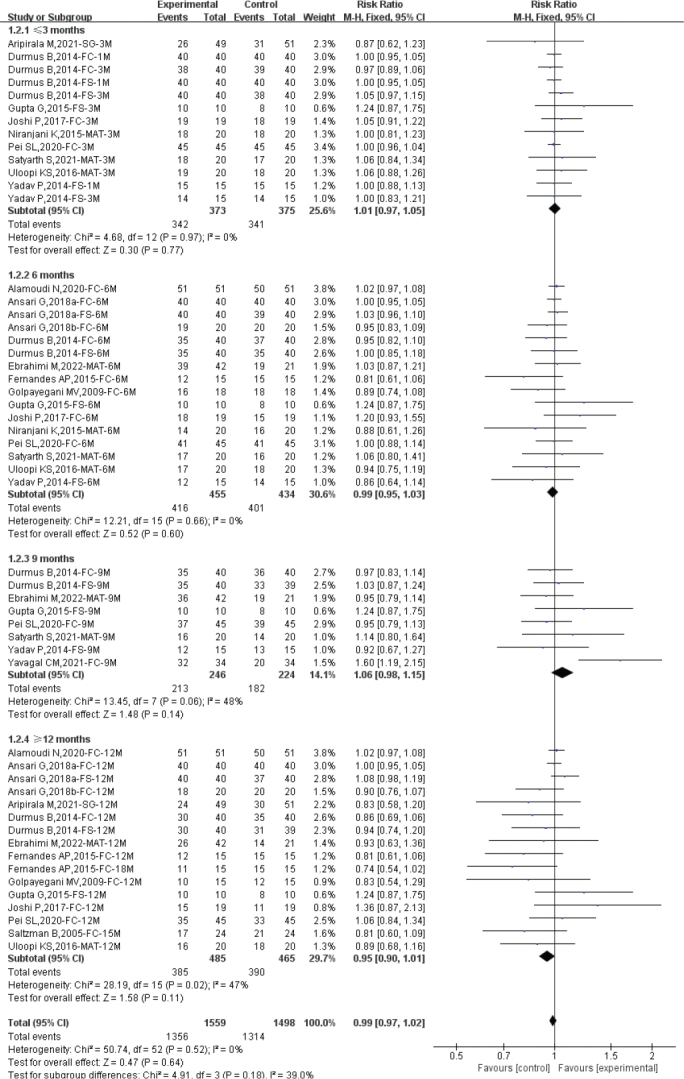
Forest plot comparing radiological success between diode laser and control by different follow-up duration.

The heterogeneity assessment showed low heterogeneity among the included studies, suggesting the use of a fixed-effect model for efficacy evaluation (Total: *I*^2^ = 0%; ≤3 months: *I*^2^ = 0%; 6 months: *I*^2^ = 0%; 9 months: *I*^2^ = 48%; ≥12 months: *I*^2^ = 47%). Overall, there was no statistically significant difference in radiographic success rates between diode laser pulpotomy and the control group (RR: 0.99; 95% CI: 0.97–1.02). Subgroup analysis by different follow-up times found that compared to the control group, diode laser pulpotomy had similar radiographic success rates at ≤3 months (*n* = 714; RR: 1.01; 95% CI: 0.97–1.05), 6 months (*n* = 889; RR: 0.99; 95% CI: 0.95–1.03), 9 months (*n* = 470; RR: 1.06; 95% CI: 0.98–1.15), and ≥12 months (*n* = 950; RR: 0.95; 95% CI: 0.90–1.01) (see Figure 5).

### Subgroup analysis by different control groups

Due to the differences in control groups used in the included studies, subgroup analyses were conducted based on different control groups to further determine whether there were significant differences in efficacy between diode laser pulpotomy and different controls.

**Figure 6:**
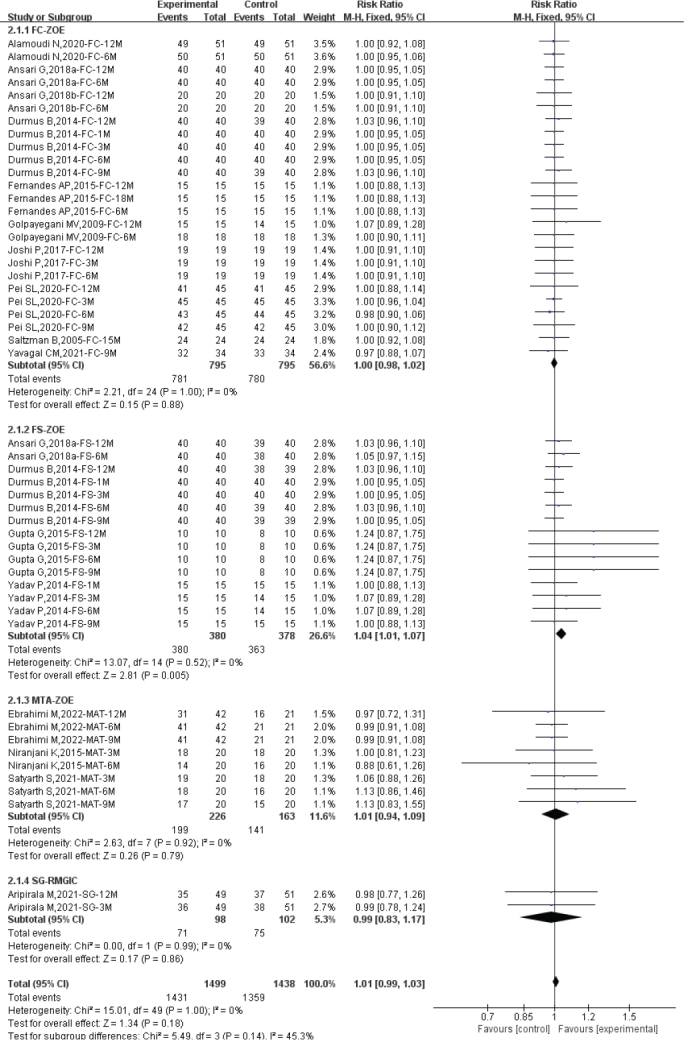
Forest plot of clinical success between diode laser and different control group.

Regarding clinical success rate, the heterogeneity assessment showed low heterogeneity among the included studies, and a fixed-effect model was used for efficacy evaluation (Total: *I*^2^ = 0%; FC-ZOE: *I*^2^ = 0%; FS-ZOE: *I*^2^ = 0%; MTA-ZOE: *I*^2^ = 0%; SG-REGIC: *I*^2^ = 0%). Compared to FC-ZOE (*n* = 1,590; RR: 1.00; 95% CI: 0.98–1.02), MTA-ZOE (*n* = 389; RR: 1.01; 95% CI: 0.94–1.09), and SG-REGIC (*n* = 200; RR: 0.99; 95% CI: 0.83–1.17), diode laser pulpotomy had similar clinical success rates. Compared to FS-ZOE, diode laser pulpotomy was more likely to achieve clinical success (*n* = 758; RR: 1.04; 95% CI: 1.01–1.07) (see Figure 6).

**Figure 7:**
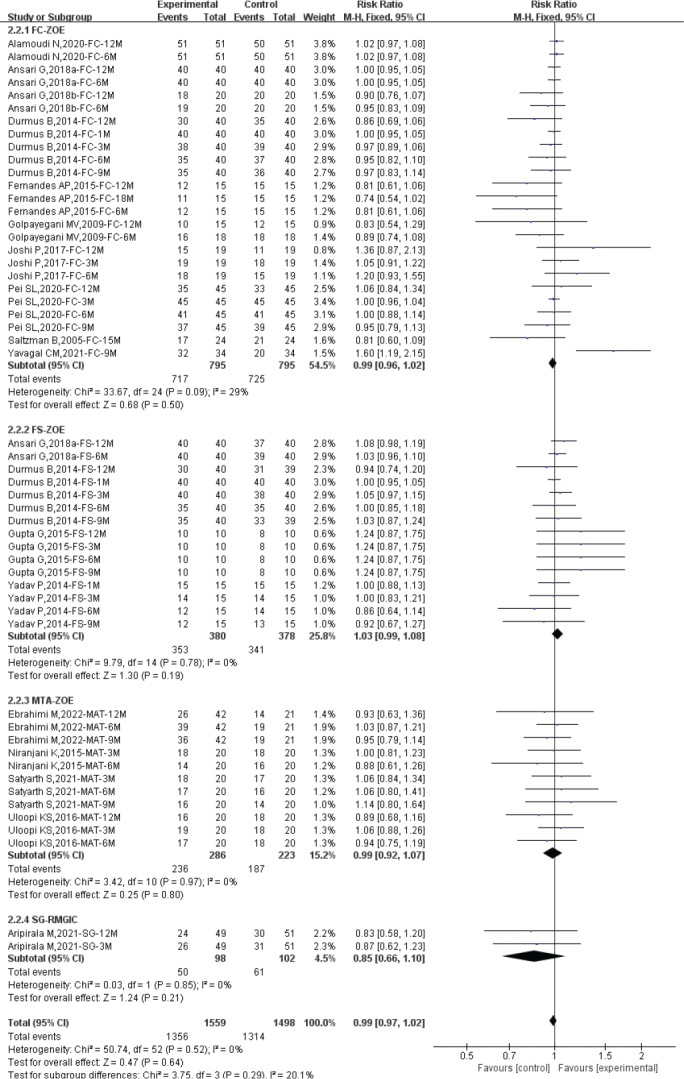
Forest plot of radiological success between diode laser and different control group.

Regarding radiographic success rate, the heterogeneity assessment showed low heterogeneity among the included studies, and a fixed-effect model was used for efficacy evaluation (Total: *I*^2^ = 0%; FC-ZOE: *I*^2^ = 29%; FS-ZOE: *I*^2^ = 0%; MTA-ZOE: *I*^2^ = 0%; SG-REGIC: *I*^2^ = 0%). Compared to FC-ZOE (*n* = 1,590; RR: 0.99; 95% CI: 0.96–1.02), FS-ZOE (*n* = 758; RR: 1.03; 95% CI: 0.99–1.08), MTA-ZOE (*n* = 509; RR: 0.99; 95% CI: 0.92–1.07), and SG-REGIC (*n* = 200; RR: 0.85; 95% CI: 0.66–1.10) (see Figure 7).

### Publication bias and certainty of evidence

**Figure 8:**
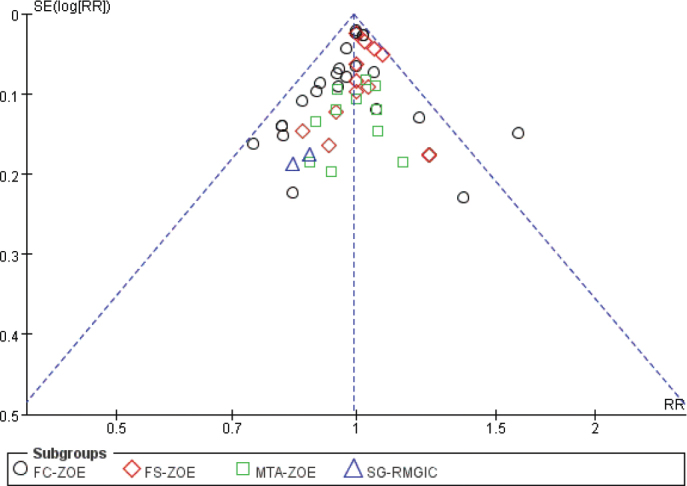
Funnel plot of included studies.

We assessed the presence of significant publication bias among the included studies using funnel plots. The results showed that the included studies were evenly distributed in the upper part of the funnel, with no significant publication bias detected (Figure 8). In addition, the results of the certainty of evidence were summarised in Supplementary Table 1. In Section 2.4, we described the contents of the bias assessment. We found that only four studies had a high risk of blinding implementation. All included studies had a low risk of bias in random allocation, allocation concealment, completeness of outcome data, selective reporting of study results, and other sources of bias (Figures 2 and 3). In addition, the heterogeneity of included studies was evaluated using *I*^2^ statistics, and all results indicated the heterogeneity was modest, with *I*^2^ ranging from 0% to 48% (most results were 0%). Therefore, we considered the included studies to have an acceptable quality in risk of bias assessment.

## Discussion

This study included current research evaluating diode laser pulpotomy in primary teeth compared to conventional pulpotomy. We used meta-analysis to comprehensively assess the clinical and radiographic success rates of diode laser pulpotomy. A total of 17 RCTs met the inclusion criteria of this study, involving 1,018 primary teeth. Follow-up results in the short term (≤3 months), medium term (6–9 months), and long term (≥12 months) all showed similar clinical and radiographic success rates between diode laser pulpotomy and conventional pulpotomy. When comparing different control groups, diode laser pulpotomy showed similar efficacy with FC-ZOE, FS-ZOE, MTA-ZOE, and SG-REGIC. However, diode laser pulpotomy demonstrated a higher likelihood of clinical success compared to FS-ZOE, suggesting potential advantages in specific clinical scenarios.

Laser-assisted pulpotomy has gained widespread acceptance because of its notable advantages over traditional methods. Diode lasers, in particular, are favoured for their reliability and simplicity. However, concerns regarding peripheral thermal damage to pulp tissue have been raised. This issue can be mitigated by optimising laser parameters such as power, frequency, and application time, as well as employing water irrigation during the procedure [29]. Some researchers recommend an irradiation time of 1–3 seconds to minimise degenerative changes [30]. For instance, Pei et al. [24] utilised diode laser parameters set at 1.5w with a 2-second irradiation time, resulting in a thin laser-induced necrotic layer that prevented direct contact with the capping material, thereby reducing chemical or toxic effects. In this study, the included studies used diode laser with output wavelengths ranging from 632 nm to 980 nm, and power settings from 10 mW to 10W, using both contact and non-contact modes. Nevertheless, the impact of these parameter variations on treatment efficacy remains unclear and warrants further investigation. Notably, Saltzman et al. [15] reported lower radiographic success rates with a 980 nm wavelength and 3W power laser compared to the FC group, primarily because of periodontal lesions and pathological root resorption. While most included studies show comparable therapeutic outcomes between diode laser pulpotomy and FC, Gupta et al. [21] found that diode laser achieved a higher success rate than FS, aligning with our findings that diode laser exhibits similar clinical and radiographic success rates to FC and MTA but significantly outperforms FS in clinical success rates.

*In vitro* studies by Cannon et al. [31] demonstrated that diode laser treatment induced milder pulp inflammation compared to FC and FS after 28 days of observation in mechanically exposed pig teeth. Yamakawa et al. [32] further supported these findings by showing that diode laser enhances alkaline phosphatase activity, promoting the formation of dentin-like cells. In addition, Sivadas et al. [33] conducted an *in vivo* study on histological changes following pulpotomy in primary teeth and found that both diode laser and FS facilitated dentin bridge formation at the pulp interface, with the diode laser group having superior dentin bridge quality. These findings collectively suggest that diode laser pulpotomy not only results in less inflammation but also stimulates the mineralisation and repair capacity, making it a relatively safe and effective treatment modality.

Despite these promising results, the current evidence remains inconclusive, highlighting the need for further high-quality RCTs with standardised protocols to establish definitive conclusions regarding the efficacy and safety of diode laser pulpotomy. Future research should focus on optimising laser parameters, assessing long-term outcomes, and comparing diode laser pulpotomy with other emerging pulpotomy techniques to provide a more comprehensive understanding of its clinical applications.

Although this study comprehensively and scientifically explored the clinical and radiographic follow-up results of diode laser pulpotomy, several limitations should be acknowledged. Firstly, some of the included studies had relatively small sample sizes, which may limit the statistical power and generalisability of the findings. Furthermore, certain studies did not implement allocation concealment and blinding, potentially introducing bias risks that could affect the reliability of the results. Another significant limitation is the heterogeneity in diode laser parameter settings across the included studies. Variations in output power, energy density, wavelength, and the mode of application (contact vs. non-contact) were observed, making it challenging to draw definitive conclusions about the optimal parameters for diode laser pulpotomy. Due to insufficient data, it was not possible to perform a detailed subgroup analysis to further investigate the relationship between specific laser parameter settings and clinical or radiographic success rates. These limitations highlight the need for future research to standardise laser parameters, employ larger sample sizes, and adhere to rigorous methodological practices, such as allocation concealment and blinding, to minimise bias and enhance the validity of the findings. Addressing these issues in future studies will provide more robust evidence to guide clinical decision-making regarding diode laser pulpotomy in primary teeth.

## Conclusion

In summary, diode laser pulpotomy in primary teeth is a viable alternative to conventional treatment methods. Compared to conventional treatment, diode laser treatment shows similar clinical and radiographic success rates in short-term (≤3 months), medium-term (6–9 months), and long-term (≥12 months) follow-ups. Therefore, diode laser pulpotomy can be considered a suitable alternative to conventional treatment. However, because of certain limitations in this study, future research should conduct more RCTs with larger sample sizes, consistent methodology, and standardised laser characteristics to further explore the clinical application efficacy of diode laser pulpotomy.

## Supplementary Material



## Declarations

### Availability of data and materials

All data generated or analysed during this study are included in this article. Further enquiries can be directed to the corresponding author.

### Registering systematic review protocols

INPLASY202540034.
