# Supplementary material for: Clinical efficacy of diode laser for pulpotomy in primary teeth: a meta-analysis of randomised controlled trials
Source: Acta Odontol Scand. 2025 Jun 10;84:43804. doi: 10.2340/aos.v84.43804 (PMC12186441; doi:10.2340/aos.v84.43804)
Supplement: Supplementary file 1 [file AOS-84-43804-s1.pdf]

Supplementary material has been published as submitted. It has not been copyedited or typeset by Acta Odontologica Scandinavica

Supplementary Table 1. GRADE evidence profile

Certainty assessment							№ of patients	Effect		Certainty
№ of studies	Study design	Risk of bias	Inconsistency	Indirectness	Imprecision	Other considerations	Success	Relative (95% CI)	Absolute (95% CI)	
17	Randomized controlled trial	not serious	not serious	not serious	not serious	not serious	1018	1.01 (0.99 to 1.03)	-	⊕⊕⊕◯ Low
**№ of studies**	**Study design**	**Risk of bias**	**Inconsistency**	**Indirectness**	**Imprecision**	**Other considerations**	**Success**	**Relative (95% CI)**	**Absolute (95% CI)**	**Certainty**
17	Randomized controlled trial	not serious	not serious	not serious	not serious	not serious	1018	0.99 (0.97 to 1.02)	-	⊕⊕⊕◯ Low
